# 48847 Assessing Transition Outcomes in Sickle Cell Disease (SCD) Prior To Implementation of A Formal Transition Program

**DOI:** 10.1017/cts.2021.726

**Published:** 2021-03-30

**Authors:** Sydney Sheppard, Julie Kanter

**Affiliations:** 1 University of Alabama at Birmingham School of Medicine; 2 Hematology/Oncology, University of Alabama at Birmingham

## Abstract

ABSTRACT IMPACT: To identify potential facilitators and barriers to a successful transition in care. OBJECTIVES/GOALS: Improvements in care for children with sickle cell disease (SCD) have increased survival into adulthood. However, mortality rates are increasing in young adults. One of the challenges is providing appropriate care during transition from pediatric to adult care. The goal is to identify facilitators and barriers to a successful transition in care. METHODS/STUDY POPULATION: The UAB SCD Center serves a large area of Alabama. The pediatric program is in Birmingham and has outreach clinics in three other cities. The adult program only has one clinic located in Birmingham. With IRB approval, we performed a retrospective chart review of individuals with SCD (all genotypes) aged 18-24 (as of 1/31/2019) who were seen at least twice prior to age 18 (in pediatrics) and have confirmed SCD. Charts were reviewed for demographics, genotype, last known insurance, SCD therapy, clinic location, and transition status. Analyses were undertaken to determine predictors of successful transition (defined as coming to an appointment with an adult hematologist) and unsuccessful transition (defined as lost to follow-up (LTFU) without transfer of care). RESULTS/ANTICIPATED RESULTS: There were 544 individuals meeting inclusion criteria. Of this group, 234 were LTFU, 189 transitioned, 36 moved, and 15 died. Seventy patients are still under pediatric care and were excluded. Sixty-eight percent of patients that transitioned had their last pediatric visit in Birmingham, compared to only 32% of those that transitioned from outreach sites (p<.01). Patients were more likely to successfully transition if they had sickle cell anemia (HbSS or HbSÃŸ0) (p<.01) and if they were receiving hydroxyurea or chronic transfusion therapy (p<.01). DISCUSSION/SIGNIFICANCE OF FINDINGS: Geography, genotype, and SCD therapy are potential drivers for transition. Genotype in pediatrics likely confers disease severity, suggesting patients with worse SCD may be more likely to successfully transition. Novel strategies are needed to improve transition of care for patients outside of Birmingham and those with less severe disease.

